# A Case of Co-infection Due to Shigella flexneri Colitis Resulting in Bacillus Septic Shock in an Immunocompetent Patient

**DOI:** 10.7759/cureus.65364

**Published:** 2024-07-25

**Authors:** Kevin T Dao, Amrit Dhillon, Syed Saad Uddin, Jose Garcia-Corella, Elias Inga Jaco, Mahum Zahid, Rupam Sharma, Hobart Lai

**Affiliations:** 1 Internal Medicine, University of California, Los Angeles (UCLA) - Kern Medical, Bakersfield, USA

**Keywords:** colitis, septic shock, immunocompetent, bacteremia, bacillus, shigella flexneri

## Abstract

Shigella flexneri (S. flexneri) is a facultatively anaerobic gram-negative bacterium that is a member of Enterobacteriaceae. The bacterium has been known to cause mild symptoms, such as diarrhea, to more severe diseases such as hemorrhagic colitis. Fortunately, such instances of severe diseases are rare. Nevertheless, even though S. flexneri is a more benign bacterium of the Shigella genus when compared to Shigella dysenteriae, this doesn’t mean that it should be neglected. In fact, the ability of this microorganism to cause inflammation of the colon or colitis and disrupt tissue architecture can allow other organisms that would otherwise be benign to cause severe complications, hence allowing said organisms to be opportunistic. Here, we would like to present a case of S. flexneri colitis resulting in bacillus bacteremia and eventually causing an inappropriate physiological host response leading to hypotension, systematic organ failure, etc., also known as septic shock. The pathogenesis and treatment of this patient will also be discussed.

## Introduction

Worldwide, the yearly incidence of Shigella (S.) is approximately 188 million, with the majority of cases presenting in the pediatric population and resulting in approximately 164,000 deaths per year, making it the second leading cause of diarrhea-associated death after rotavirus [1]. In many low-socioeconomic countries, S. flexneri is the most common bacterium of the Shigella genus, composing approximately 65.9% of Shigella isolates [2]. Fortunately, most Shigella species, such as S. flexneri, are generally self-limiting in immunocompetent patients. As a result, the average length of symptoms associated with Shigella-related gastroenteritis tends to be one week. Unfortunately, like many other bacteria, antibiotic resistance tends to be a main issue, allowing the microorganism to persist and cause a wide range of symptoms. In fact, Shigella dysenteriae type 1 has been shown to cause more severe symptoms that can result in a high fatality rate [3]. S. flexneri has also been reported to cause severe disease, ranging from mild symptoms, such as diarrhea, to more severe pathologies like necrotizing hemorrhagic colitis [4]. Some studies have reported that S. flexneri has a wide range of conditions, including acute colitis [5,6]. This, in turn, can allow opportunistic organisms to invade the bloodstream by creating microperforations in the colon, allowing various pathogens to cause an inappropriate physiological response, also known as septic shock. In this case, the patient was noted to have a bacterial bloodstream infection, also known as bacteremia, which is a curiosity since patients with Bacillus bacteremia tend to have other co-morbidities. One study documented 11 cases of Bacillus bacteremia in a six-year time span and said patients had cancer as an underlying disease [7]. Thus, we would like to discuss in detail how the pathogenesis of both of these bacteria resulted in our immunocompetent patient developing septic shock, as well as our approach to diagnosis and treatment.

## Case presentation

The patient is a 53-year-old female who presented to the emergency department (ED) with a complaint of diffuse abdominal pain for the past 4 days. The patient reported symptoms of abdominal cramping with approximately three to four episodes of non-bloody, watery diarrhea and non-bloody emesis. She also noted that her symptoms started after eating old, canned chicken soup along with some rice for dinner. The patient stated that she also had some subjective fevers but no other symptoms. Of note, she also denied any sick contacts or recent travel.

**Figure 1:**
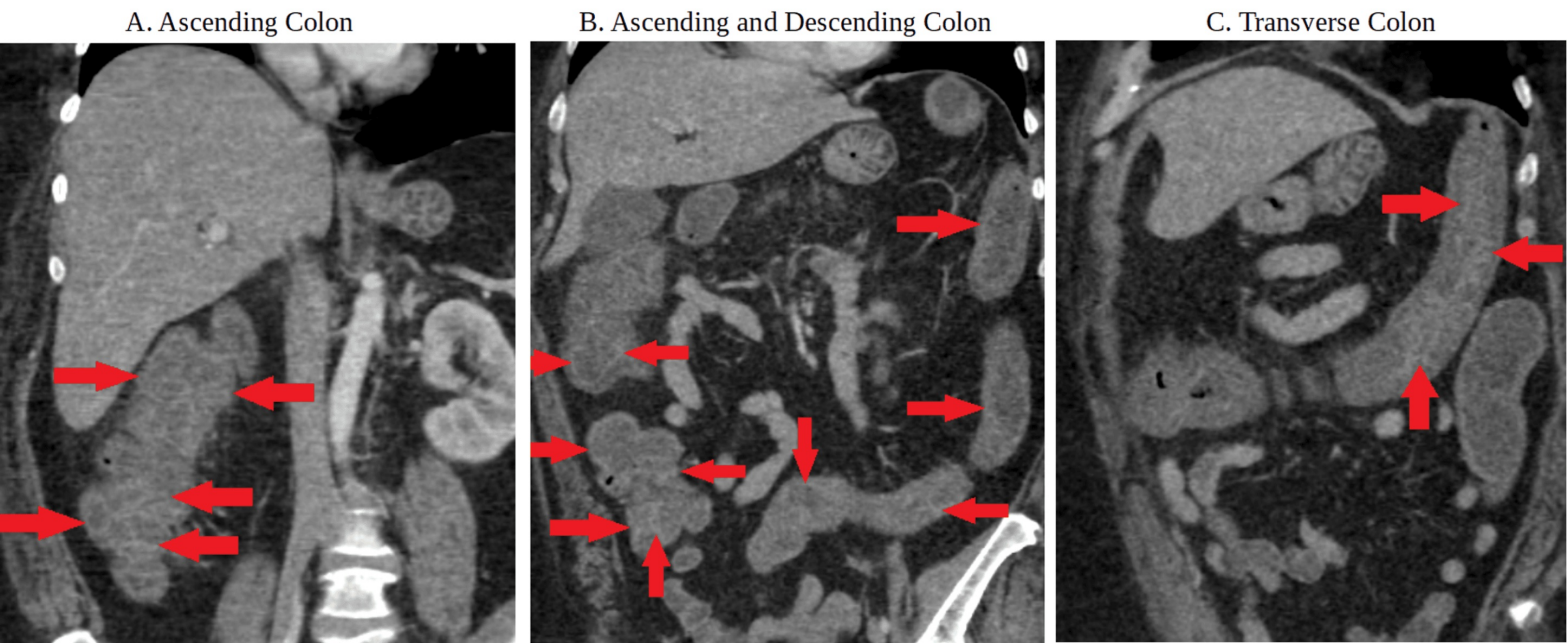
Computed tomography abdomen and pelvis with contrast There is circumferential, moderate, wall thickening of the ascending, transverse, and descending colon consistent with colitis with the red arrows delineating contrast being trapped between edematous haustral folds also known as the “accordion” sign.

In the ED, the patient was noted to be tachycardic, with a heart rate in the 120s to 130s. Her systolic blood pressure was in the 60s and her diastolic blood pressure was in the 40s, with a mean arterial pressure in the 50s. The patient's rectal temperature was also 39.5 °C. The rest of the physical exam reveals diffuse abdominal tenderness to superficial palpation with mild guarding but otherwise unremarkable. Blood work depicted a lactic acid level of 5.1 mmol/L and a white blood count (WBC) of 18.6 x 10^9/L with 42% bands. Basic metabolic panel (BMP) and complete blood count (CBC) were otherwise unremarkable. HIV was negative, and the patient was not taking any immunosuppressive medications. Blood cultures were drawn, the patient was given 4L of intravenous fluids, started on vancomycin and ceftriaxone, and was immediately admitted to the intensive care unit (ICU) for concerns of septic shock. In the ICU, the patient was only noted to have mild improvement; as such, ceftriaxone was transitioned to piperacillin/tazobactam, and the patient was started on a norepinephrine drip. Fortunately, she stabilized, although she still had complaints of abdominal pain while producing mild watery diarrhea. Stool cultures were taken, and computed tomography (CT) of the abdomen and pelvis with contrast showed diffuse mural wall thickening of the colon, suggestive of colitis (Figures 1A-1C). The patient was slowly weaned off vasopressors, and once vitals remained stable, she was downgraded to the primary care floor.

**Figure 2:**
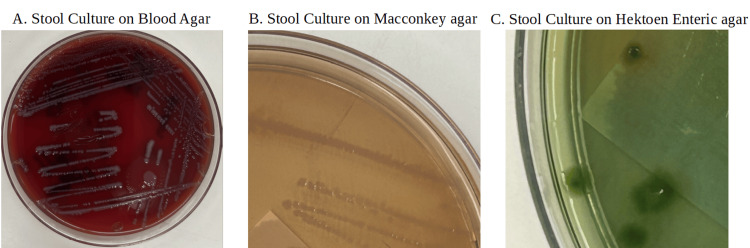
Stool cultures A. Stool culture on blood agar; B. Stool culture on Macconkey agar is shown to be non-pink, which indicates that the bacterium is noted to be non-lactose fermenting; C. Stool cultures are plated on Hektoen Enteric agar, which displays green colonies. This means that the bacterium plated doesn’t produce hydrogen sulfide, implying Shigella spp.

**Figure 3:**
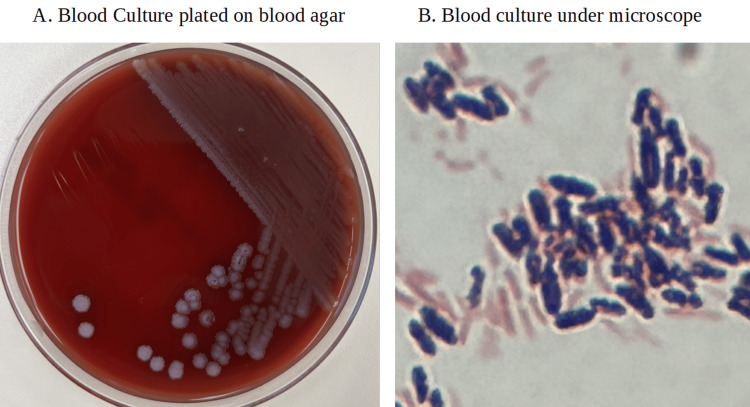
Blood culture A. Blood culture plated on blood agar; 3B. The patient’s blood cultures, which show gram-positive rods seen under the microscope

Stool cultures and speciation were positive for S. flexneri (Figures 2A-2C), and blood cultures grew positive for the Bacillus genus (Figures 3A-3B). Unfortunately, speciation could only rule out that the gram-positive rod was not Bacillus anthracis but was unable to speciate further. Piperacillin/tazobactam was discontinued, and the patient was started on ceftriaxone for Shigella and continued on vancomycin for bacillus bacteremia. After repeat cultures were negative and the patient remained stable, she was eventually discharged with oral trimethoprim-sulfamethoxazole for 10 days.

## Discussion

Based on the patient’s history, blood work, cultures, and imaging, it was concluded that the patient developed S. flexneri colitis, resulting in Bacillus bacteremia, which eventually progressed to septic shock. This is interesting, firstly given how shigellosis is expected to be found in the pediatric population, men who have sex with men, and travelers to endemic low and middle-income countries [1], and secondly, because of the development of superimposed bacteremia from a different pathogen. Thus, its presentation in a healthy, middle-aged female is unusual. Regardless, once the Shigella bacterium was identified prompt treatment with third-generation cephalosporin, macrolide, trimethoprim-sulfamethoxazole, or fluoroquinolone should be done accordingly, with susceptibility testing to ensure proper antibiotic treatment. For Bacillus-related food poisoning, antibiotics are not necessary; however, this is not the case for bacteremia. In most situations, vancomycin would be highly recommended, and our patient showed a positive response to the antibiotic.

Among different Shigella strains, S. dysenteriae tends to be more associated with colitis and toxic megacolon [8]. However, other cases have reported that S. flexneri is capable of causing these symptoms as well, with one study noting S. flexneri’s ability to cause colonic perforation [9]. This is mainly because Shigella can cause enterocolitis due to the exudative loss of various immuno-proteins such as complement and immunoglobulins [10]. This, in turn, decreases the amount of opsonization and lysis of various microorganisms, which can pertain to bacteremia and septicemia [11]. Regardless, initial management should consist of stabilizing the patient with broad-spectrum antibiotics based on the suspicion of septic shock. More specific antibiotics should then be started based on cultures. While the patient was being managed appropriately, the patient’s diagnosis of Shigella-induced pancolitis allowing another pathogen to cause septic shock was also an interesting theory. In some instances, there have been reports of other microbes causing colitis and, as a result, bacteremia [12-14] though those cases stemmed from one microbe causing the majority of the affliction. However, this is one of the few cases where other opportunistic microorganisms took advantage and seeded into the bloodstream. The fact that this was due to a Bacillus bacterium was also interesting since various forms of members of the Bacillus genus tend to affect children, immunocompromised patients with indwelling catheters, contaminated hospital equipment, or other co-morbidities [15-17]. Although this is a unique case, it is not completely unheard of. In actuality, there have been very few cases where Shigella has allowed other bugs to cause a wide host of pathologies. One study noted a co-infection of the colon in an immunocompetent patient with Cytomegalovirus and Shigellosis. The study hypothesized that the mucosal damage caused by the Shigella infection predisposes the colon to a Cytomegalovirus infection [18]. This brings up a unique point that even self-limiting infections can cause debilitating pathologies, especially when in the presence of another bacterium. This study also brings up an interesting fact about how relatively harmless bacterial species can cause severe sepsis when given the opportunity, and even immunocompetent patients without any severe co-morbidities can still be susceptible.

## Conclusions

Despite S. flexneri being a very common infection worldwide, it is generally a rare disease in the United States and the adult population, with Shigella sonnei being more commonplace. This may cause a decrease in concern among healthcare providers since the majority of Shigella infections tend to be self-limiting in immunocompetent individuals. In this case, our patient could have resolved the Shigella infection if it wasn’t also associated with a Bacillus infection. This primarily shows the possibility that some self-limiting infections can result in some instances, and they shouldn’t be ignored. Hopefully, by increasing awareness of the capability of self-limiting infections, more information can be provided as to the dangers that some of these infections can pose.
